# “What choice do you have knowing your child can’t breathe?!”: Adaptation to Parenthood for Children Who Have Received a Tracheostomy

**DOI:** 10.1177/23779608241245502

**Published:** 2024-04-09

**Authors:** Ellinor Rydhamn Ledin, Andrea Eriksson, Janet Mattsson

**Affiliations:** 1 Department of Ergonomics - CBH, 7655 KTH Royal Institute of Technology, Huddinge, Sweden; 2 Department of Health Sciences, Swedish Red Cross University, Huddinge, Sweden; 3 Department of Nursing and Health Sciences, University of South-Eastern Norway, Campus Vestfold, Norway; 4 4342 Department of Nursing and Integrated Health Sciences, Kristianstad University, Kristianstad, Sweden

**Keywords:** pediatrics, neonatology, intensive care unit, tracheostomy, parenthood

## Abstract

**Introduction:**

A growing number of parents are navigating parenthood influenced by medical complexity and technological dependency as the group of children with long-term tracheostomy grows. However, little is known regarding the parental experiences of parenthood for this heterogeneous group of children now surviving through infancy and intensive care.

**Objective:**

This study aimed to analyze how parents of children who have received a tracheostomy adapted to parenthood.

**Methods:**

Interviews were conducted and analyzed following a constructivist grounded theory approach. Ten parents of seven children living with a tracheostomy in Sweden were recruited via the long-term intensive care unit (ICU).

**Results:**

The core variable of parenthood “Stuck in survival” was explained by two categories and six subcategories. The category “Unaddressed previous history” describes the experiences from being in the ICU environment and how the parents are not able, due to insufficient time and resources, to address these stressful experiences. The category “Falling through the cracks of a rigid system” describes how the parents found themselves and their children to be continuously ill-fitted in a medical system impossible to adapt to their needs and situation. Parents placed the starting point of parenthood with the birth of the child, whilst the tracheotomy only constituted a turning point and would lead to the loss of any previously held expectations regarding parenthood.

**Conclusion:**

This study identified a previously undescribed period prior to tracheostomy placement, which may have long-lasting effects on these families. The care provided in ICUs following the birth of a child who will require tracheostomy may not be tailored or adapted to accommodate the needs of these families leading to long-lasting effects on parenthood.

## Introduction

The parallel medical and technological developments have made survival possible for more children with diseases or conditions affecting their airway organs and/or breathing, thereby giving rise to a change (Carron et al., 2000; Gergin et al., 2016), growing and heterogeneous group of technology-dependent children (Brenner et al., 2021) living with long-term tracheostomy (Liu et al., 2014; Watters, 2017). The increased survival and transfer of care from hospital to home affects parents as it assumes that parents will provide complex and critical care on top of parenthood. Previous research suggests that balancing parenthood with providing complex and technology-dependent care affect parents as well as parenthood itself (Flynn et al., 2013; Kirk et al., 2005). However, little is known about the current everyday lives and experiences of parenthood for the parents of these children, and in order to reach beyond survival and ensure good and equal living conditions, resources, and research must be allocated to investigate and address the actual problems, hindrances, and struggles faced by these families. Therefore, the purpose of this study was to analyze how parents of children who have received a tracheostomy adapted to parenthood.

## Review of Literature

The indication for tracheostomy has shifted since the 1970s from airway obstruction due to infectious diseases (Carron et al., 2000), towards congenital conditions and/or complications following extremely premature birth (Gergin et al., 2016). Previous research regarding children with tracheostomy has focused on singular events during hospitalization, starting with the decision to place a tracheostomy (Hebert et al., 2017), the surgery and outcomes affecting in-hospital care (Campisi & Forte, 2016; Davidson et al., 2021; D'Souza et al., 2016; Okonkwo et al., 2020), or the transition from hospital to home (Amar-Dolan et al., 2020). For a child to be discharged with a tracheostomy, some form of home nursing care or assistance is needed to uphold and sustain everyday life and living conditions for the child and their family. Globally, details, routines, and practices of in-hospital care, hospital discharge, and home care may differ. Initiatives to improve the quality and safety of tracheostomy care as well as to improve the quality of life for persons with tracheostomy have been undertaken. However, while such initiatives are needed to structure and standardize care, the focus and centricity may risk missing the ethical considerations and dilemmas of granting survival but failing to provide the prerequisites for everyday life and quality of life throughout the life of a child. Globally, children with tracheostomy are a group with high and complex needs, in both short- and long-term, in hospital, and out of hospital.

Dependency on a greater number of medical devices during neonatal intensive care unit (NICU) hospitalization has been shown to result in greater family burden (Grunberg et al., 2020). Previous research also points out that children's technology dependence alters parents’ perception and the construction of parenthood (Kirk et al., 2005). While a tracheostomy can lead to survival and add to an expanding group of children living with tracheostomy, as many as 72% of parents have been reported to regret letting their child undergo tracheotomy as soon as three months after surgery (Hebert et al., 2017). Parents of children with tracheostomy face specific challenges related to their caregiving experiences, social experiences, and experiences of service organization and delivery of care (Flynn et al., 2013). Therefore, the purpose of this study was to analyze how parents of children who have received a tracheostomy adapted to parenthood, specifically focusing on the initial months spent in hospital following birth as that would be the time were the foundation of parenthood is formed.

## Methods

### Design, Qualitative Approach, and Research Paradigm

This study used the constructivist grounded theory [C-GT] (Charmaz, 2014) approach. This design was chosen to reach the experiences from the perspective, construction, and understanding of parents and to yield a theoretical framework grounded in data derived from interviews with the parents as there is limited previous research on the matter. Parenthood can be considered a social construct which further motivated the choice of design. C-GT can be considered to adhere to the interpretivism/constructivism paradigm, with a relativist ontology and a constructivist epistemology (Urcia, 2021).

The reporting methods used were based on the Standards for Reporting Qualitative Research, SRQR (O'Brien et al., 2014).

### Researcher Characteristics and Reflexivity

Interviews were conducted by two interviewers, both female and with previous experience of conducting interviews. The interviewers’ background, their reasons for conducting the study, as well as information about the project, was stated at the beginning of all interviews.

### Context and Sampling Strategy

Participants were recruited via a long-term intensive care unit (ICU) in a larger city in Sweden, where children could receive tracheostomy care on in- or out-patient basis (regardless of the family's area of residence). If treated on an out-patient basis the children were cared for at home by their parents and with the support of personal caregivers trained in tracheostomy care. If still receiving in-patient care, care was provided by parents and hospital staff.

Inclusion criteria were parents of children who currently were or had been tracheotomized and able to communicate in English and/or Swedish. Information regarding the study was posted on the unit's bulletin board as well as sent to parents of out-patient tracheotomized children with the aid of a nurse in the unit. Parents were encouraged to contact the research team if they were interested in participating in the study and were then invited to schedule an interview at a time and date of the parent's convenience.

A convenience sample consisting of all parents who articulated a willingness to participate was established. As there were no registers describing the demographics or total number of children currently living with long-term tracheostomy any other form of sampling was considered unattainable. The convenience sample consisting of 10 parents (of seven children living with long-term tracheostomy) were interviewed individually from June to October 2021, either in person (3) or via Zoom (7) depending on the preference of the parent. One parent did not show up for the scheduled interview which was considered as a desire to drop out of the study.

### Ethical Considerations and Issues Pertaining to Human Subjects

Ethical approval was obtained from the Swedish Ethical Review Authority (reference number 2017/1722-31-1) as a part of a larger research project. The entirety of the study has been planned and conducted in compliance with the declaration of Helsinki (World Medical Association, 2013). Written and verbal informed consent to participate was collected prior to the interview. Reassurance of the parents’ confidentiality and that their participation would not interfere with or affect the care the child was receiving at the unit was written in the information and repeated at the start of the interview. Due to the small size of the sample and the subsequent risk of identification of names and genders in excerpts that have been edited, extra attention has also been given to the presentation of characteristics of the transcript excerpts to maintain participant confidentiality. All transcripts and recordings were stored locally on a password-protected hard drive.

### Data Collection Methods, Instruments, and Technologies

Interviews were chosen to reach parents’ experiences in depth. Interviews allowed for flexibility, adaptability, and possibilities to ask parents to elaborate or share in-depth details of their experiences (McGrath et al., 2019). Due to the unexplored nature of the research area and focus, all interviews were made sure to cover the general experience of receiving medical care and a set of areas (tracheostomy, interaction, technology dependence, relationships, parental role, and child's way of communication) in the form of a topic guide. The topic guide was piloted, and the outcome was assessed and qualified to be included for analysis. No changes were made to the guide following the pilot test. Other topics that the parents considered important or chose to bring up during the interviews were always adhered to and parents were asked to expand on. This method yielded rich-detailed and open-ended interviews, which all began with the question “How did it come about that you child now has a tracheostomy?”.

The interviews were recorded, either audio only (in-person interviews) or video and audio (interviews via Zoom), and stored locally on the recording device. The interviews lasted between 60 and 195 minutes dependent on when the interviewed parent felt that they had shared all they wished to share and address, in total 12 hours and 29 minutes. Due to a technological error in Zoom, the recordings of four interviews were not saved and transcription was not possible. Notes taken during these four interviews were supplemented with details recalled by both interviewers and used as a form of theoretical sampling.

### Units of Study

In total 10 parents, five mothers and five fathers, were interviewed. Two of the 10 parents had encountered adults with long-term tracheostomy because of their occupation within healthcare. However, none of the parents had friends, family, or relatives who were tracheostomy dependent. All parents, except for one father, were still in the relationship where the child was conceived. Six of the in total seven children were treated on an out-patient basis and one child was still hospitalized and received care on an in-patient basis. Most parents held a job outside of the home, except for one mother who was full-time caring for the child in the hospital and two mothers who full-time cared for their child at home. This was the first surviving child for all parents. Three of the children were girls and four were boys. The underlying condition differed between the children and none of the children had the exact same set of diagnoses. Three of the children were born extremely premature (weeks 22–23) and had bronchopulmonary dysplasia, however, they also had additional diagnoses such as pulmonary hypertension, tracheomalacia, or cerebral palsy. The remaining four children were born at term and were diagnosed with bilateral vocal cord paralysis, genetic syndrome, and craniofacial abnormalities after a tumor and one child was still undiagnosed. Parents interviewed and the corresponding additional characteristics of the children are described in Table 1.

**Table 1.** Interviewed Parents and Corresponding Child Characteristics.

Parent interviewed	Age (at tracheostomy)	Age (at interview)	Ventilator	Oxygen
Mother, father	3 weeks	8 years	No	No
Mother, father	6 months	1.5 years	Yes	Yes
Mother, father	4 months	3 years	Yes	Yes
Mother	5 months	3 years	Intermittent	No
Mother	3 months	3 years	No	No
Father	Under 6 months	12 years	No	No
Father	Under 6 months	3 years	Yes	Yes

### Data Analysis and Processing

The recorded interviews were transcribed verbatim by the two interviewers and stored locally on password-protected hard drives. In cases where notes were taken during the interviews, the notes were saved as separate files and stored together with the corresponding transcript. To maintain participant confidentiality no transcripts nor fieldnotes were shared until names had been redacted by the transcriber. Data saturation was discussed within the research team and deemed sufficient for analysis.

The transcripts were subjected to iterative coding and analysis following C-GT (Charmaz, 2014) which can be simplified in the following processes:

1. Initial coding was performed by *line-by-line coding* each transcript.
2. Codes derived from line-by-line coding are analyzed and recoded during *open coding.* Here similarities can be identified between the different interviews and prominent features within the dataset started to emerge.
3. Open codes were then analyzed during *focused coding*, and categories and relationships between codes and categories were identified.
4. Codes and categories are formed into the emerging theoretical framework during *theoretical coding*.

This step-by-step, but iterative, analysis process slowly raised the analytical and abstract levels and allowed for codes, categories, and relationships to emerge freely from the data without force or use of pre-formed theories. Whilst some argue that themes can be formed through grounded theory analysis (Morse, 2008) we followed a C-GT (Charmaz, 2014) and how the term category is used throughout the reference literature. Ideas regarding the analysis and the emergent theoretical framework were written in *memos* during the entire analysis and coding process. Transcripts, notes, and memos were revisited to further inform the theory during the final stages of analysis. Examples and excerpts from the analysis are presented in Table 2.

**Table 2.** Examples From Analysis.

Quote	Subcategory	Main category	Core variable
…It was like a nightmare that just went on and on and on and never ended … so you had to pick yourself up right away … wake up, onward, no baby bubble here… **I3** … lack of oxygen for 9 minutes, so we were sure [child] had brain damage too, but it was nothing. Amazing… **I1**	Suffering a traumatic and adverse start	**Unaddressed previous history**	**Stuck in survival**
…because she was so sick, so she didn't have any energy to do things or play, and these other needs didn't exist in the same way … she didn't really have the energy for them… **I6** …you keep hoping that removing the trach will be the thing that will … remove everything, but it won’t. [Child] still has a syndrome, [child] still late in some things … we don't know if [child] will go to a normal school, we don't know if [child] will be able to easily understand everything, we don't know if [child] will have a job, we don't know if [child] will have a [relationship or partner], we don't know if … [child] will have children, so we don't know anything. So that part still makes you sad… **I3**	Enduring the ever-present medical severity	**Unaddressed previous history**	**Stuck in survival**
…it feels even more because when you have a sick child, then it becomes almost like a plague … I don't really know what to say, they don't really know how to act or how they should talk about your child … It becomes lonely… **I3** … I know that we were informed about a closed Facebook group but I'm like this no no no, I don't have Facebook and I can’t handle the social or like no then it was easier for me somehow to just try to handle it yourself, so it's a bit … the flip side of it is that it's like constantly reinventing the wheel in some way like that, how to do this situation… **I2**	Solo-navigating uncertainty and complexity	**Unaddressed previous history**	**Stuck in survival**
… you’re always between two points, between chairs … it is sad like that you’re in this situation, frustration that you are in this situation because you’re not in one place you’re in two different places… **I4** …what they already think maybe that … if this doesn't happen then maybe we have to resort to place a trach … then maybe I could also see it [the trach] as a way out and wouldn’t have to think that my child will die because nothing can be done the entire time … it [the trach] is the last resort and then maybe you still have … it more as a positive thing maybe in the end rather than having [the trach] as a negative thing. Because what you thought was like we try, and we try and now suddenly it became a trach in the end anyway … but why should he have a trach when we didn't even try that much before… **I3**	Lack of collaboration and co-ordination of care	**Falling through the cracks of a rigid system**	**Stuck in survival**
…like a trust that's one thing then you start doubting you start all over you know in the field every time you talk about something and then you start question is that's you know, do I need to ask or something? Are they really making the right decision? That's one of the things I think I have … kind of like side effects that I had from those things… **I5**	Parents (non)participation in their children's care	**Falling through the cracks of a rigid system**	**Stuck in survival**
…“We are going to have to do a tracheostomy” We’re like “okay!,” cause, what choice do you have knowing your child can’t breathe?! … so “yeah, okay” … within three weeks [of age] [child] had a tracheostomy yeah and then life change. You think having a child is lifechanging but having your new child with a situation like this it's a … a different level. And then it was fine, [child] could breathe, so it was better but worse so to speak… **I4**	A decision but not a choice	**Falling through the cracks of a rigid system**	**Stuck in survival**

### Techniques to Enhance Trustworthiness

The common criteria for trustworthiness in qualitative research are credibility, dependability, confirmability, transferability (Lincoln & Guba, 1985), and authenticity (Guba & Lincoln, 1994). The interviews rendered a rich and substantial amount of data aided by the topic-guide and letting the interviewed parents decide when all relevant subjects and topics had been addressed. The topic-guide supported the collection of rich and in-depth data through upholding structure and similarity between interviews, yet it allowed for flexibility and exploration. The iterative analysis and coding process was repeatedly discussed within the entire research team allowing for analyst triangulation. Withholding from an initial review of literature prior to analysis, as is praxis in C-GT (Charmaz, 2014), was foundational to reach a result and form a theoretical framework grounded in data from the interviews.

## Results

This result presents an early period in the journey of parents to children living with tracheostomy, starting from the birth of the child, and ending with the turning point constituted by the tracheostomy. All children presented with airway and breathing difficulties from birth and would remain in-hospital until receiving the tracheostomy.

Six subcategories emerged which could be sorted into two categories: *unaddressed previous history* and *falling through the cracks of a rigid system. Stuck in survival* emerged as the core variable and characterized parenthood for these parents.


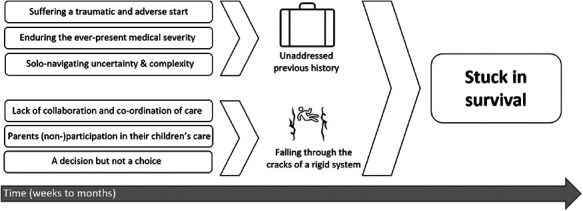


### Unaddressed Previous History

Parents experienced a pattern of stressful and potentially traumatic events heavily influenced by the uncertainty of the child's survival. Experiences were carried unaided and unresolved leading to their preservation and encapsulation, thereby influencing, and coloring the experience of parenthood. The unaddressed history contributed to a medicalized and institutionalized parental role formed by hospital care and hospital staff.

### Suffering a Traumatic and Adverse Start

Parents experienced that the birth of the child did not play out as expected and were surprised that the child survived birth without contracting severe brain damage. Parents of children born at term described their children as being born “unresponsive” or “stillborn” and the children were often accompanied by complications contracted during birth. Parents of children born preterm described how symptoms of an imminent preterm birth had been explained as normal and nothing to worry about, thereby contributing to a delay in seeking medical care and limiting the possibilities for proper care in relation to premature birth.

Following birth and resuscitation, intensive care was initiated. The terms of the ICU’s physical environment were set by the hospital staff and their routines and practices. The ICU environment was perceived to demand consideration for other families by sitting still bedside, keeping one's voice down, and refraining from using smartphones. The possibility of taking pictures or recording videos of the newborn could be lost and forgotten owing to this demand. Remaining bedside when the newborn was physically unstable could be challenging. Being in the ICU meant not being able or allowed to hold the newborn. The usual and expected activities with a newborn simply were not possible to conduct with this newborn and contributed to a forced distance between parent and child. Overlooking the parents’ own health and physical symptoms was described as something that any parent would do in a situation when the health and survival of their child were threatened.

…we were always in this emergency room scenario type thing. So, we would see people come in in of various sorts of states, children in various sort … We personally were sort of veterans, we’ve been there for like, we’ve spent three months in hospital which again is a different you get institutionalized… **I4**

### Enduring the Ever-Present Medical Severity

Complex and technologically advanced procedures were conducted to manage the symptoms and/or determine the child's underlying condition. The complexity and severity of the condition led to a long time spent in-hospital divided between multiple units and, at times, even multiple hospitals. A focus on pathology, symptoms, and the medical care of the child rendered the care in general to become driven by disease. Sensing the child's medical severity and complexity before staff noticed or acknowledged it was frequently described. When parents were not listened to, but rather deflected, a tear in the trust of the staff involved emerged.

The care received in ICU was communicated by staff to the parents in real time, and parents experienced that this was done without addressing potential prognosis or plan forward. The view of the staff guided care and interventions for the child. Care received was described as reactive rather than proactive, and decisions regarding the child's medical care were formed later rather than sooner. The child therefore had to suffer severe deterioration before a change in care would be decided for.

…in NICU after she passed the most critical moments then we can see that … actually it is false image, but we think that [child] is developing well… **I5**

Arriving at a diagnosis, if possible, took substantial amounts of time and left the parents to endure long periods of uncertainty and ongoing medical severity. Naming a specific diagnosis or condition was reached in conjunction with the child not responding as expected to the medical care given and therefore subjected to further testing or screening. This contributed to parental struggles to understand and retain an overview of the child's care and condition.

…they don't really know why this happened and why [child] has so many deformities. No explanation at all, and they have also performed genetic tests … but they found nothing. **I1**

### Solo-Navigating Uncertainty and Complexity

Parents described frequent feelings of isolation and loneliness. However, bringing visitors into the ICU was not always allowed, leaving vulnerable parents attached to staff instead. Staff were considered and often referred to as family or friends. Parents often described an unwillingness to act in a way that could portray them as annoying or troublesome parents in front of staff.

Supportive interventions to manage the parents’ situation were described as suboptimal as they were non-accessible, non-existent, or not tailored to the parents’ needs. Parents who did receive support often received it from a counselor at the hospital and a set number of occasions. Support could be offered through the hospital priest regardless of the religious views of the family. Some parents found reassurance via social media by seeing children with tracheostomy living their everyday lives, just like any other child.

…the frustration and the anger and the sadness and that's always there and then never leaves you, you just get used to it … And you deal with it the best you can as I said I’ve been in therapy, drink, drugs, hypnotherapy, and therapy, still in therapy … yeah it is solid it is difficult, really difficult … but you know you do what you can… **I4**

Being transferred between units or hospitals could mean profound insecurity for parents, with no one being able to determine the duration of receiving care elsewhere. Thoughts regarding the death of the child would resurface when informed of transfers due to escalation of care or the need for immediate intervention.

Parents could be left as the only ones situated to see the child behind the medical complexity when staff focused solely on pathology. Parents could then face challenges in integrating the dual perspective of a child and a medically complex patient, as well as the dual roles of being a parent and a medical caregiver.

### Falling Through the Cracks of a Rigid System

Parents described how, due to the complexity and uncertainty of their children's underlying conditions, their children did not fit into the care given in one medical unit alone. As a result, parents and children required care in a way that did not suit the healthcare system as is, and the system did not seem to have enough flexibility to adapt to the needs of the families.

### Lack of Collaboration and Co-ordination of Care

The challenges for parents in fulfilling multiple roles were, for some parents in the study, a highly physical experience and a collision of routines and practices between units. Hospital routines and practices were often built on a model of prioritizing medical needs and the belief in a cure, similarly, hospitalization as well as medical needs were considered and modeled as transitory. Routines were described as demanding separation in situations where a medical need was prominent in both the child and the birthing parent.

… [child] was one of the sickest kids in twenty years’ time in [unit], things like that but nobody tells us, it just pop-out from like casual talk, we would like to know that, how sick [child] was and I think we are entitled to know, but nobody really said that. So, a lot of times it feels like they tried to make things sounds easier… **I5**

Turmoil could arise when transferring between units especially if the child would deteriorate or the medical needs would change ever so slightly. Wrongful transfers and de-escalation of care were described as the result of the decision-making staff not yet understanding or grasping the child's medical complexity and/or severity.

An alienation of parents from information and decisions regarding care was described as a form of one-way communication where staff would control the information and parents held a function as data sources. Individual staff members would decide which information was important enough to relay, which some parents viewed as a measure of protection. Parents frequently described a lack of information and a sense of not being let in on the plans, often leading to suspicions about a hidden agenda and plans long gone ahead but not shared. Parents described how discussions with staff present at birth or from other ICUs had been canceled or forgotten, and how they themselves had to seek up staff to discuss what had transpired. This way of handling information would contribute to the struggle for parents to grasp and understand their children's care and condition by positioning the needs of staff in the foreground, and the needs of parents in the background.

Information to parents from staff regarding tracheostomy placement was frequently described as coming out of nowhere and as a final “hail Mary” when all other interventions had failed. Most parents had no previous understanding or knowledge of tracheostomies, tracheostomy care, or the impact a tracheostomy have on everyday life. Misconceptions were common among parents and often indicated an underestimation of needs and demands for adequate tracheostomy care. Parental misconceptions or concerns were rarely perceived as addressed or solved by the staff. Feelings of fear, inadequacy, and overwhelming responsibility were present among parents when presented with information regarding a tracheostomy.

### Parents (Non)Participation in Their Children's Care

Parental presence and participation in the child's care were described as fluctuating between units, with medical needs, and with current technological dependence. Staff's expectation of how much care parents should independently conduct would fluctuate with higher medical needs leading to lower expectations of parental participation and independence.

…when we move to [unit] they didn’t even use the machine before and we can see that and know it, and that was the most difficult time … we were NOT able to participate in rounds and we can see some of the people don’t get update of something we think are important and have been repeatedly happened … and on the other hand there's not enough data that I could check by myself, so I don’t think that's good… **I5**

Parental participation during rounds differed between units and depended on the current units’ practices. Parents were expected to follow and adopt current practices regardless of their previous experiences. Parents described being moved further away from rounds over time although they experienced that staff expected them to independently conduct more of their children's care with time. Rounds were perceived as important because they were an arena for decisions and plans. Not being present or not participating could therefore leave parents with a sense of not having insight into their child's care and doubts whether their child received the best available care. Decisions made during rounds could therefore risk portraying a false process where parents looked participatory when decisions were, in fact, made behind closed doors by staff alone.

…we ended up there [specific unit] because they knew that the last resort is trach, and it will be soon. And now it's just to get like … working on the parents that we need to have a trach… **I3**

The parental role in the decision to tracheostomy was frequently described as one of pretend and that parents had no choice but to prepare for what was coming. The decision was perceived to have been made by the staff before approaching the parents. Gaining parental consent seemed to be the focus, rather than parental participation in the actual decision. Inactive participation in the decision was fueled by information from the staff that they would place the tracheostomy without consent if needed. Several parents described that the staff's threshold for placing a tracheostomy seemed low, and interventions prior to asking for parental consent were described as insufficient, lacking, or not robust enough.

### A Decision But Not a Choice

Parental consent to tracheostomy was not perceived as something possible to abstain from since the child would not be able to breathe and thereby survive without the tracheostomy. The mere days between giving consent and surgery highlighted and illustrated a new and fast-paced process in their children's care.

Parents giving their consent for tracheostomy were described in three different paths. The first was through *uncomplicated acceptance*; consent was given almost immediately as the information was presented to the parents and not questioned or challenged. Parents described trusting the multiple experts involved. The second path was *considering an option:* a path where the parents would weigh the option of remaining in the ICU, with another breathing support until the child was stable, against a tracheostomy. However, most parents vent down the third path, a path of *persuasion*, where staff in various ways tried to persuade and get parents to give consent. Attempts to persuade the parents were ineffective and described by parents in terms of foul, ugly, and sneaky. These negative experiences occurred regardless of the form or media of information regarding tracheostomy placement.

The reason for parents to give their consent, across all pathways, were the physical factors parents had seen in the child, most prominently the child's struggle to breathe. Some parents considered an increased physical growth a positive side effect of the tracheostomy by reducing energy consumption and the labor of breathing.

…But it wasn't that they asked us … it was quite clear that we thought it was difficult … but they presented it as [child] needed it and then you can't say so much … [child] was in such bad shape then … So, if you're going to have a trach, then it should be because you need it, it shouldn't be that you could choose … if you can choose, you do not need it. **I6**

Placing the tracheostomy constituted a turning point in parenthood, as a child with a tracheostomy and the care needed was new to the parents and would demand their full attention from there onwards. The turning point meant a shift of focus and redirection from the child surviving their underlying condition to surviving with a tracheostomy. This shift would form and shape parenthood to mimic and retain the roles given to parents during the initial ICU hospitalizations prior to the child receiving the tracheostomy. Parenthood ended up being forced to remain *stuck in survival*.

## Discussion

This study aimed to analyze how parents of children who have received a tracheostomy adapted to parenthood. These parents were found to endure both an *unaddressed previous history* and were continuously *falling through the cracks of a rigid system*. The core variable of parenthood *stuck in survival* can be interpreted as a parental frame of mind formed during the ICU hospitalization and retained after the turning point of the child receiving the tracheostomy.

In this study, it has been shown that the formation of parenthood for these parents starts with the birth of the child, not with the tracheostomy. By not recognizing this parental perspective, it may continue to risk creating trauma through providing a poorly adapted and tailored care. A multitude of experiences during the period from birth to tracheostomy have the potential to become traumatizing, especially since parents in this study have described unattainable and inaccessible psychosocial support. The results of this study are in stark contrast to trauma-informed care (TIC) that could aid in mitigating the risk of ill-effects of trauma and the development of postintensive care syndrome, posttraumatic stress symptoms, and/or pediatric medical traumatic stress (Demers et al., 2022). However, a parenthood continuously *stuck in survival* would eradicate the experience of a “post,” and rather pointing towards ongoing and continuous experiences from the birth of the medically complex and technology-dependent child. An area for future research could be parents of children with other forms of technology-dependence as they may have similar experiences.

Intensive care has been defined as the management, monitoring, and treatment of patients with or at risk of life-threatening organ dysfunction, focusing on the underlying pathology and solving organ dysfunction rather than the underlying illness or condition (Marshall et al., 2017). This could be considered similar to how the tracheostomy has been proposed as a time-giving “bridge” (Flanagan & Healy, 2019) to support the airway organs while directing treatment toward the underlying condition. However, the comparison raises the question of whether living with a tracheostomy should be considered a continuous form of intensive care. However, a tracheostomy should be considered lifesaving and life-sustaining indications of complex and severe underlying conditions and their correct management of critical importance. It is therefore not surprising that the parents would redirect and shift their focus from the child surviving the underlying condition to survival with a tracheostomy after the turning point of tracheotomy as presented in the results of this study.

While the centricity of care and view of children have undergone changes during the past decades, these children may be in a situation where such changes may have been more challenging to implement (Butler et al., 2014). Centricity of care in high-dependence or ICUs may be harder to change due to the severe illness and high technological dependence, thereby increasing the risk of a “dehumanization” of the ICU-patient (Velasco Bueno & La Calle, 2020; Wilson et al., 2019). Rules and practices from the unit or hospital may also contribute and act as barriers (Baird et al., 2015).

This study describes a lack of shared information as well as a lack of shared decision-making, both in opposition to how family centered care (FCC) (Butler et al., 2014) is described. These aspects may contribute to the challenges these parents face to receive support for their parenthood and not just for the medical- and nursing care they provide. Upholding all aspects of FCC (Butler et al., 2014) could hold further significant importance by allowing for parent–child interaction by enabling parental presence in ICUs and ensuring accessible and attainable support for parents of hospitalized children. Human interactions, especially parent–child interactions, are of utmost importance to support the normal and/or expected developmental trajectory of the child and should be explored further regarding children with tracheostomy. Parents are essential for the well-being and thriving of children; perhaps even more so under circumstances of hospitalization and medical needs, parental well-being is therefore of utmost importance to support and maintain. Medical and technological developments that grant survival for more complex conditions are not likely to diminish, more families in similar situations are therefore likely to be encountered in the future. The sustainable development goals state that “…healthy lives and promote well-being for all at all ages” (United Nations, 2015) should be ensured. Therefore, merely settling for survival should not be considered “good enough” for either parents or children.

Families being cared for in ICUs may be perceived as “difficult” by staff (Ashana et al., 2020; Baird et al., 2015), not due to their personal behavior, but perhaps more so as a sign of not fitting the current medical system or contextual factors, thereby creating friction (Baird et al., 2015). This emergent friction could risk unintentionally forming certain units to act as “catch-all” for medically complex and high-dependence children without sufficient specialization or a high enough level of care. The choice of where to treat families at risk of such friction could play a pivotal role in how neonatal ICUs, pediatric ICUs, and pediatric units in general are designed and planned.

There seems to be little or no conflict between parents and medical care providers regarding the child's need for the tracheostomy in this study. Rather, the conflict and struggles seem to be regarding the process and way the decision was made. This notion would render the decision to place a tracheostomy and its process rather different from seemingly similar decisions of withholding or continuing life support, where the decision itself is the source of conflict (Spijkers et al., 2022). While previous research has thoroughly investigated the tracheostomy decision (Bushroe et al., 2023) or caregiving experiences in relation to transitioning from hospital to home with a tracheostomy (Callans et al., 2016), this study may contribute by addressing influential factors prior to such events and how they affect parenthood.

### Strengths and Limitations

This study is not without limitations. Seven families constitute a relatively small sample, and saturation cannot be stated with complete certainty. However, the results of this study are in line with previous research on parental experiences but provide additional details, aspects, and in-depth descriptions of their experiences. By using the four interviews affected by the technological error to test the final framework against, as a form of theoretical sampling, it can be argued that this managed to strengthen the trustworthiness and rigor (Lincoln & Guba, 1986) of the framework but may have missed details brought up specifically during those four interviews. Recruitment of additional participants was difficult because of the timeframe as well as having included all parents who expressed an interest to participate. However, the heterogeneity and diversity of the children's characteristics may speak for a broad and diverse set of parental experiences.

Even if the children's characteristics were heterogeneous and diverse, there was a strong consensus among parents regarding multiple aspects, as presented in the results. The risk of the results being a local experience during this early period prior to tracheostomy seems small as the seven children were born and treated at different units. The movement towards centralization of certain types of treatments, care, and/or interventions could also be argued to decrease the risk of us identifying local conditions.

The three different paths to parental consent could for example be considered an indication that the richness and detail of the data have been preserved and maintained throughout the analysis and that the potential loss of data or critical details from the four interviews affected by the technological error had small to minimal effect on the end result. These aspects can be considered to strengthen the overall trustworthiness and rigor (Lincoln & Guba, 1986) of the study.

Experiences of parents who declined tracheostomy, parents of children who have passed away, or parents of children who are decannulated are not included in the results of this study and should be considered for further research.

### Implications for Practice

These results imply that there is a need to adjust and tailor current care and practice to suit the growing group of parents to the now surviving children dependent on long-term tracheostomy. More emphasis and focus could be directed towards adopting Family Centered and Trauma Informed Care in neonatal and pediatric ICUs to support the developing parenthood of these parents and move beyond mere survival. These results also highlight the need for continuous and tailored parental psychosocial support as well as a more transparent dialogue and discussion regarding the tracheostomy decision process.

## Conclusions

This study identifies a previously undescribed but important period for parents of children who are dependent on tracheostomy. Adaptation to parenthood was identified as being stuck in survival, meaning that the role and mission of these parents is to ensure their child's survival, first through the underlying condition and thereafter with a tracheostomy. This would consolidate and retain the role parents and parenthood had during the initial period spent in ICUs from the birth of the medically complex and technology-dependent child.

## References

[bibr1-23779608241245502] Amar-Dolan L. G. Horn M. H. O'Connell B. Parsons S. K. Roussin C. J. Weinstock P. H. Graham R. J. (2020). “This Is How Hard It Is”. Family experience of hospital-to-home transition with a tracheostomy. Annals of the American Thoracic Society, 17(7), 860–868. 10.1513/AnnalsATS.201910-780OC 32267725 PMC7328176

[bibr2-23779608241245502] Ashana D. C. Lewis C. Hart J. L. (2020). Dealing with “Difficult” patients and families: Making a case for trauma-informed care in the intensive care unit. Annals of the American Thoracic Society, 17(5), 541–544. 10.1513/AnnalsATS.201909-700IP 31944818 PMC7193814

[bibr3-23779608241245502] Baird J. Davies B. Hinds P. S. Baggott C. Rehm R. S. (2015). What impact do hospital and unit-based rules have upon patient and family-centered care in the pediatric intensive care unit? Journal of Pediatric Nursing, 30(1), 133–142. 10.1016/j.pedn.2014.10.001 25450441 PMC4405525

[bibr4-23779608241245502] Brenner M. Alexander D. Quirke M. B. Eustace-Cook J. Leroy P. Berry J. Healy M. Doyle C. Masterson K. (2021). A systematic concept analysis of ‘technology dependent’: Challenging the terminology. European Journal of Pediatrics, 180(1), 1–12. 10.1007/s00431-020-03737-x 32710305 PMC7380164

[bibr5-23779608241245502] Bushroe K. Crisp K. Politi M. Brennan S. Housten A. (2023). Evaluating caregiver-clinician communication for tracheostomy placement in the neonatal intensive care unit: A qualitative inquiry. Journal of Perinatology: Official Journal of the California Perinatal Association. Advance online publication. 10.21203/rs.3.rs-2869532/v1 PMC11014892 37833495

[bibr6-23779608241245502] Butler A. Copnell B. Willetts G. (2014). Family-centred care in the paediatric intensive care unit: An integrative review of the literature. Journal of Clinical Nursing, 23(15-16), 2086–2099. 10.1111/jocn.12498 24372988

[bibr7-23779608241245502] Callans K. M. Bleiler C. Flanagan J. Carroll D. L. (2016). The transitional experience of family caring for their child with a tracheostomy. Journal of Pediatric Nursing, 31(4), 397–403. 10.1016/j.pedn.2016.02.002 27040188

[bibr8-23779608241245502] Campisi P. Forte V. (2016). Pediatric tracheostomy. Seminars in Pediatric Surgery, 25(3), 191–195. 10.1053/j.sempedsurg.2016.02.014 27301607

[bibr9-23779608241245502] Carron J. D. Derkay C. S. Strope G. L. Nosonchuk J. E. Darrow D. H. (2000). Pediatric tracheotomies: Changing indications and outcomes. The Laryngoscope, 110(7), 1099–1104. 10.1097/00005537-200007000-00006 10892677

[bibr10-23779608241245502] Charmaz K. (2014). Constructing grounded theory (2nd ed.). Sage Publications.

[bibr11-23779608241245502] Davidson C. Jacob B. Brown A. Brooks R. Bailey C. Whitney C. Chorney S. Lenes-Voit F. Johnson R. F. (2021). Perioperative outcomes after tracheostomy placement among complex pediatric patients. The Laryngoscope, 131(8), E2469–e2474. 10.1002/lary.29402 33464608

[bibr12-23779608241245502] Demers L. A. Wright N. M. Kopstick A. J. Niehaus C. E. Hall T. A. Williams C. N. Riley A. R. (2022). Is pediatric intensive care trauma-informed? A Review of Principles and Evidence. Children (Basel), 9(10), 1575. 10.3390/children9101575 PMC9600460 36291511

[bibr13-23779608241245502] D'Souza J. N. Levi J. R. Park D. Shah U. K. (2016). Complications following pediatric tracheotomy. Jama Otolaryngology-- Head & Neck Surgery, 142(5), 484–488. 10.1001/jamaoto.2016.0173 27055048

[bibr14-23779608241245502] Flanagan F. Healy F. (2019). Tracheostomy decision making: From placement to decannulation. Seminars in Fetal & Neonatal Medicine, 24(5), 101037. 10.1016/j.siny.2019.101037 31699570

[bibr15-23779608241245502] Flynn A. P. Carter B. Bray L. Donne A. J. (2013). Parents’ experiences and views of caring for a child with a tracheostomy: A literature review. International Journal of Pediatric Otorhinolaryngology, 77(10), 1630–1634. 10.1016/j.ijporl.2013.07.020 23953483

[bibr16-23779608241245502] Gergin O. Adil E. A. Kawai K. Watters K. Moritz E. Rahbar R. (2016). Indications of pediatric tracheostomy over the last 30 years: Has anything changed? International Journal of Pediatric Otorhinolaryngology, 87, 144–147. 10.1016/j.ijporl.2016.06.018 27368463

[bibr17-23779608241245502] Grunberg V. A. Geller P. A. Patterson C. A. (2020). Infant illness severity and family adjustment in the aftermath of NICU hospitalization. Infant Mental Health Journal, 41(3), 340–355. 10.1002/imhj.21848 32057129

[bibr18-23779608241245502] Guba E. G. Lincoln Y. S. (1994). Competing paradigms in qualitative research. In Handbook of qualitative research (pp. 105–117). Sage Publications, Inc.

[bibr19-23779608241245502] Hebert L. M. Watson A. C. Madrigal V. October T. W. (2017). Discussing benefits and risks of tracheostomy: What physicians actually say. Pediatric Critical Care Medicine, 18(12), e592–e597. 10.1097/pcc.0000000000001341 PMC5716895 28938289

[bibr20-23779608241245502] Kirk S. Glendinning C. Callery P. (2005). Parent or nurse? The experience of being the parent of a technology-dependent child. Journal of Advanced Nursing, 51(5), 456–464. 10.1111/j.1365-2648.2005.03522.x 16098162

[bibr21-23779608241245502] Lincoln Y. S. Guba E. G. (1985). Naturalistic inquiry. Sage.

[bibr22-23779608241245502] Lincoln Y. S. Guba E. G. (1986). But is it rigorous? Trustworthiness and authenticity in naturalistic evaluation. New Directions for Program Evaluation, 1986(30), 73–84. 10.1002/ev.1427

[bibr23-23779608241245502] Liu C. Heffernan C. Saluja S. Yuan J. Paine M. Oyemwense N. Berry J. Roberson D. (2014). Indications, hospital course, and complexity of patients undergoing tracheostomy at a tertiary care pediatric hospital. Otolaryngology—Head and Neck Surgery, 151(2), 232–239. 10.1177/0194599814531731 24788698

[bibr24-23779608241245502] Marshall J. C. Bosco L. Adhikari N. K. Connolly B. Diaz J. V. Dorman T. Fowler R. A. Meyfroidt G. Nakagawa S. Pelosi P. Vincent J. L. Vollman K. Zimmerman J. (2017). What is an intensive care unit? A report of the task force of the World Federation of Societies of Intensive and Critical Care Medicine. Journal of Critical Care, 37, 270–276. 10.1016/j.jcrc.2016.07.015 27612678

[bibr25-23779608241245502] McGrath C. Palmgren P. J. Liljedahl M. (2019). Twelve tips for conducting qualitative research interviews. Medical Teacher, 41(9), 1002–1006. 10.1080/0142159X.2018.1497149 30261797

[bibr26-23779608241245502] Morse J. M. (2008). Confusing categories and themes. Qualitative Health Research, 18(6), 727–728. 10.1177/1049732308314930 18503013

[bibr27-23779608241245502] O'Brien B. C. Harris I. B. Beckman T. J. Reed D. A. Cook D. A. (2014). Standards for reporting qualitative research: A synthesis of recommendations. Academic Medicine, 89(9), 1245–1251. 10.1097/acm.0000000000000388 24979285

[bibr28-23779608241245502] Okonkwo I. Cochrane L. Fernandez E. (2020). Perioperative management of a child with a tracheostomy. BJA Educ, 20(1), 18–25. 10.1016/j.bjae.2019.09.007 33456911 PMC7807921

[bibr29-23779608241245502] Spijkers A. S. Akkermans A. Smets E. M. A. Schultz M. J. Cherpanath T. G. V. van Woensel J. B. M. van Heerde M. van Kaam A. H. van de Loo M. Willems D. L. de Vos M. A. (2022). How doctors manage conflicts with families of critically ill patients during conversations about end-of-life decisions in neonatal, pediatric, and adult intensive care. Intensive Care Medicine, 48(7), 910–922. 10.1007/s00134-022-06771-5 35773499 PMC9273549

[bibr30-23779608241245502] United Nations (2015). *Goals, 03. Ensure healthy lives and promote well-being for all at all ages.* Retrieved 2023-05-30 from https://sdgs.un.org/goals/goal3.

[bibr31-23779608241245502] Urcia I. A. (2021). Comparisons of adaptations in grounded theory and phenomenology: Selecting the specific qualitative research methodology. International Journal of Qualitative Methods, 20, 16094069211045474. 10.1177/16094069211045474

[bibr32-23779608241245502] Velasco Bueno J. M. La Calle G. H. (2020). Humanizing intensive care: From theory to practice. Critical Care Nursing Clinics of North America, 32(2), 135–147. 10.1016/j.cnc.2020.02.001 32402312

[bibr33-23779608241245502] Watters K. F. (2017). Tracheostomy in infants and children. Respiratory Care, 62(6), 799–825. 10.4187/respcare.05366 28546379

[bibr34-23779608241245502] Wilson M. E. Beesley S. Grow A. Rubin E. Hopkins R. O. Hajizadeh N. Brown S. M. (2019). Humanizing the intensive care unit. Critical Care, 23(1), 32. 10.1186/s13054-019-2327-7 30691528 PMC6350326

[bibr35-23779608241245502] World Medical Association (2013). Declaration of Helsinki: Ethical principles for medical research involving human subjects. Jama, 310(20), 2191–2194. 10.1001/jama.2013.281053 24141714

